# In-Hospital Cardiac Arrest in the Cardiac Catheterization Laboratory: Effective Transition from an ICU- to CCU-Led Resuscitation Team

**DOI:** 10.1155/2019/1686350

**Published:** 2019-09-02

**Authors:** Rajat Sharma, Hilary Bews, Hardeep Mahal, Chantal Y. Asselin, Megan O'Brien, Lillian Koley, Brett Hiebert, John Ducas, Davinder S. Jassal

**Affiliations:** ^1^Section of Cardiology, Department of Internal Medicine, Max Rady College of Medicine, Rady Faculty of Health Sciences, University of Manitoba, Winnipeg, Manitoba, Canada; ^2^Section of Critical Care, Department of Internal Medicine, Max Rady College of Medicine, Rady Faculty of Health Sciences, University of Manitoba, Winnipeg, Manitoba, Canada; ^3^Institute of Cardiovascular Sciences, St. Boniface Albrechtsen Research Centre, University of Manitoba, Winnipeg, Manitoba, Canada; ^4^Department of Radiology, Max Rady College of Medicine, Rady Faculty of Health Sciences, University of Manitoba, Winnipeg, Manitoba, Canada; ^5^Department of Physiology and Pathophysiology, Max Rady College of Medicine, Rady Faculty of Health Sciences, University of Manitoba, Winnipeg, Manitoba, Canada

## Abstract

**Objectives:**

(1) To examine the incidence and outcomes of in-hospital cardiac arrests (IHCAs) in a large unselected patient population who underwent coronary angiography at a single tertiary academic center and (2) to evaluate a transitional change in which the cardiologist is positioned as the cardiopulmonary resuscitation (CPR) leader in the cardiac catheterization laboratory (CCL) at our local tertiary care institution.

**Background:**

IHCA is a major public health concern with increased patient morbidity and mortality. A proportion of all IHCAs occurs in the CCL. Although in-hospital resuscitation teams are often led by an Intensive Care Unit- (ICU-) trained physician and house staff, little is known on the role of a cardiologist in this setting.

**Methods:**

Between 2012 and 2016, a single-center retrospective cohort study was performed examining 63 adult patients (70 ± 10 years, 60% males) who suffered from a cardiac arrest in the CCL. The ICU-led IHCAs included 19 patients, and the Coronary Care Unit- (CCU-) led IHCAs included 44 patients.

**Results:**

Acute coronary syndrome accounted for more than 50% of cardiac arrests in the CCL. Pulseless electrical activity was the most common rhythm requiring chest compression, and cardiogenic shock most frequently initiated a code blue response. No significant differences were observed between the ICU-led and CCU-led cardiac arrests in terms of hospital length of stay and 1-year survival rate.

**Conclusion:**

In the evolving field of Critical Care Cardiology, the transition from an ICU-led to a CCU-lead code blue team in the CCL setting may lead to similar short-term and long-term outcomes.

## 1. Introduction

In-hospital cardiac arrest (IHCA) is a major public health burden with increased patient morbidity and mortality. Data from large registries within the United States and United Kingdom report that the incidence of an IHCA ranges from 0.6 to 2.7 per 1000 hospital admissions [1–3]. Despite improvements in survival over the last decade, outcomes from IHCA remain poor with less than 40% of individuals surviving to hospital discharge [4–7].

Approximately 1.3% of all patients who undergo coronary angiography experience an IHCA, with 3.0% of all IHCAs occurring in the cardiac catheterization laboratory (CCL) [8, 9]. Advanced cardiac life support (ACLS) in the CCL has advantages and challenges that include the following: (i) complex coordinated team approach between the in-hospital resuscitation and catheterization lab teams with selection of a single team leader; (ii) availability of continuous physiological monitoring including ECG parameters, pulse oximetry, and invasive hemodynamics; (iii) working around fluoroscopic imaging equipment while shielding resuscitation team members from ionizing radiation; and (iv) immediate access to a mechanical LUCAS device for uninterrupted chest compressions [9–11]. Most IHCAs in the CCL resolve with brief cardiopulmonary resuscitation (CPR) and defibrillation, resulting in high procedural survival [8].

In academic centers across North America, in-hospital resuscitation teams are often led by an Intensive Care Unit- (ICU-) trained physician and house staff with the rare involvement of a cardiologist. Similarly, in the setting of an IHCA in the CCL, cardiologists are infrequent members of the CPR team [12]. In a nationwide Denmark study, cardiologists only attended 27% of all IHCAs and were the resuscitation team leader in 12% of the cases [12]. In a complementary study from Italy, cardiologists participated in only 16% of hospital resuscitation teams [13]. As a member of the cardiac arrest team, a cardiologist may possess a unique skillset, which includes cardiac rhythm analysis, bedside focus echocardiography, pericardiocentesis, and/or temporary pacing.

Given the paucity of data on the role of a dedicated cardiologist in the management of IHCAs, the objective of the current study was 2-fold: (1) to examine the incidence and outcomes of IHCAs in a large unselected patient population who underwent coronary angiography at a single tertiary academic center and (2) to compare a transitional change in which the cardiologist is positioned as the CPR leader in the CCL at our local tertiary care institution.

## 2. Methods

### 2.1. Study Population

Between 2012 and 2016, a single-center retrospective cohort study was performed, examining adult patients who suffered from a cardiac arrest while admitted to the CCL. The ICU was responsible for responding to all cardiac arrests in the CCL from 2012 to 2014. The Coronary Care Unit (CCU) assumed this role from 2015 to 2016. We identified a total of 63 patients between 2012 and 2016 who suffered an IHCA in the CCL. The ICU-led IHCAs (February 2012–December 2014 inclusive) included 19 patients, while the CCU-led IHCAs (January 2015–August 2016) included 44 patients. Only the IHCAs where resuscitation responded to a code blue were included in the study.

### 2.2. Definitions

A cardiac arrest was defined as a sudden cessation of cardiac function, precipitated by ventricular tachycardia (VT), ventricular fibrillation (VF), pulseless electrical activity (PEA), or asystole requiring CPR. ICU-led IHCA team members included a critical care specialist and critical care fellow, 2-3 residents (postgraduate trainees with surgical, medical, or anesthesia backgrounds), 2-3 registered ICU nurses, and a respiratory therapist. The CCU-led IHCA team included a cardiologist and cardiology fellow, 1 resident (postgraduate trainee with surgical, medical, or anesthesia background), an anesthesia specialist, 2-3 registered ICU nurses, and a respiratory therapist. During the study span of 2012–2016, the 2010 and 2015 AHA guidelines for CPR were utilized [14, 15].

### 2.3. Demographics

Demographic information including age, sex of patient, cause of presentation, and comorbidities were collected using a detailed chart review. Resuscitation details included cause and total duration of IHCA, standard CPR (<15 minutes), prolonged CPR (≥15 minutes), use and duration of manual or mechanical compressions, initial rhythm, procedure performed during/after cardiac arrest, and associated laboratory parameters. Primary outcomes included CCL survival, length of hospital stay, and 1-year survival rate. The secondary outcome of neurological recovery was recorded for all patients surviving the initial arrest.

### 2.4. Statistics

Baseline characteristics were listed as mean values ± standard deviation (SD) or median values (quartile 1–quartile 3) for continuous variables and percentages (absolute numbers) for categorical variables. Baseline characteristics, resuscitation results, and clinical outcomes were compared between the ICU-led and CCU-led IHCAs. Continuous variables were compared using an independent Student's *t*-test or Mann–Whitney *U*-test where appropriate. Categorical variables were compared using the Fisher exact test. Kaplan–Meier survival curves were generated to visualize the 1-year survival rate of each cohort and compared using a log-rank test. Version 9.3 of SAS was used for the statistical analysis, and *p* < 0.05 was considered statistically significant.

## 3. Results

**Figure 1:**
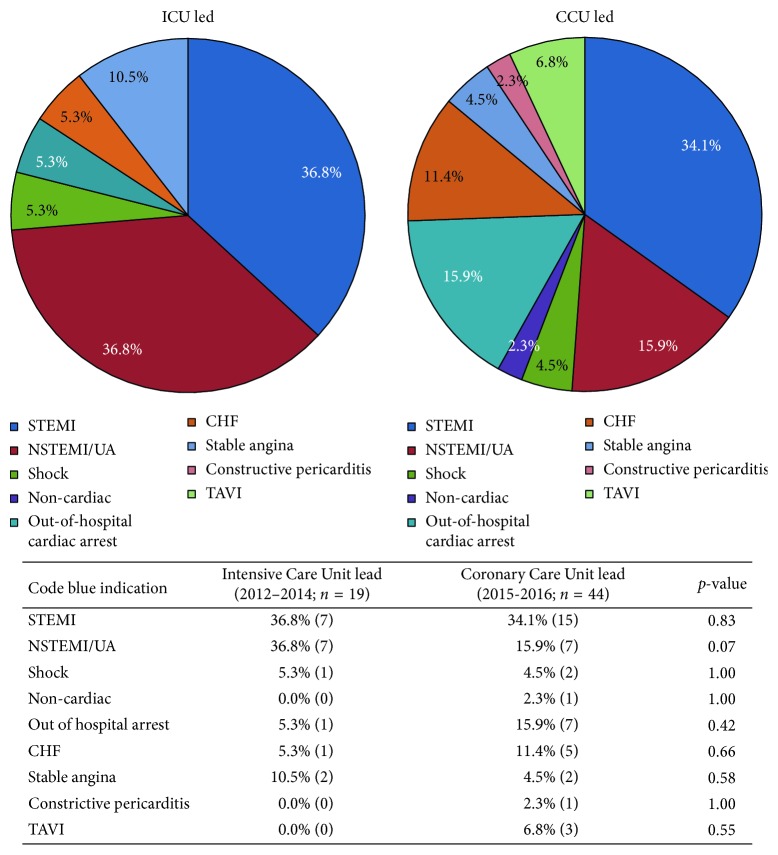
Indication for cardiac catheterization. CCU, Coronary Care Unit; ICU, Intensive Care Unit; STEMI, ST elevated myocardial infarction; NSTEMI, non-ST elevated myocardial infarction; UA, unstable angina; CHF, congestive heart failure; TAVI, transcatheter aortic valve implantation.

At our single tertiary care centre, a total of 63 (0.5%) out of 13,112 patients who underwent a coronary angiogram in the CCL suffered from an IHCA between February 2012 and August 2016. ACLS was led by the ICU team in 19 cases (2012–2014) and the CCU team in 44 cases (2015-2016). Baseline demographics of the study population are outlined in Table 1. The mean age of the study population was 67 ± 10 years in the ICU-led cohort and 70 ± 11 years in the CCU-led cohort. Cardiac risk factors including hypertension, diabetes, and hypercholesterolemia were prevalent in both groups. A significantly higher proportion of patients had a history of smoking exposure in the ICU-led cohort than in the CCU-led cohort (53% vs. 18%; *p* < 0.01). ST elevation myocardial infarction (STEMI) was a common indication for coronary angiography in up to 1/3 of the study population (Figure 1). Non-STEMI (NSTEMI) or unstable angina constituted the second most common indication for coronary angiography, representing 37% and 16% of the ICU-led and CCU-led cohorts, respectively (*p*=0.07). Other indications for coronary angiography included cardiogenic shock, out-of-hospital cardiac arrest (OHCA), congestive heart failure (CHF), stable angina, right heart catheterization, and noncardiac indications (Figure 1).

ACLS was initiated in the CCL for PEA more frequently than ventricular arrhythmias and/or hypotension (Table 1). In the ICU-led group, a total of 3/19 (16%) patients received standard CPR and 16/19 (84%) patients received prolonged CPR (≥15 minutes). In the CCU-led group, a total of 12/44 (27%) patients received standard CPR and 32/44 (73%) patients received prolonged CPR (≥15 minutes). Although the total length of CPR was similar in both groups, standard CPR (<15 minute duration) was shorter in the CCU-led cohort. The majority of patients received chest compressions, including mechanical chest compressions via a LUCAS device in over 25% of the patient population. The duration of manual and mechanical CPR was similar between the ICU-led and CCU-led cohorts (Table 1). Nearly 2/3 of patients required further respiratory support and were subsequently intubated. Mechanical circulatory support (MCS) using an Impella ventricular assist device was frequently employed in both the ICU-led and CCU-led cohorts (39% versus 21%; *p*=0.13).

The cause of hemodynamic deterioration was most commonly attributed to cardiogenic shock in the ICU-led cohort as compared to the CCU-led cohort (84% vs. 23%; *p* < 0.01) as shown in Table 1. Conversely, an arrhythmogenic cause for cardiovascular collapse was more frequent in the CCU-led cohort as compared to the ICU-led cohort (39% vs. 5%; *p* < 0.01). The rate of coronary dissection was similar between the two cohorts. Finally, a minority of patients in the CCU-led cohort suffered from a left ventricular free wall rupture, pulmonary embolus, or pulmonary arterial rupture.

Approximately 50% of the study population received emergent revascularization by percutaneous coronary intervention (PCI). In the ICU-led cohort, 10/19 (53%) patients underwent simultaneous PCI during the IHCA, of which 3 (16%) individuals received mechanical compressions with a LUCAS device. In the CCU-led cohort, 21/44 (48%) patients underwent simultaneous PCI during resuscitation, of which 3 (7%) individuals also received mechanical compressions. A greater proportion of patients in the ICU-led cohort, as compared to the CCU-led cohort, was surgically revascularized by coronary artery bypass surgery (22% vs. 7%; *p*=0.18). Emergent pericardiocentesis or valvular surgery was necessary in a minority of cases (Table 1).

**Figure 2:**
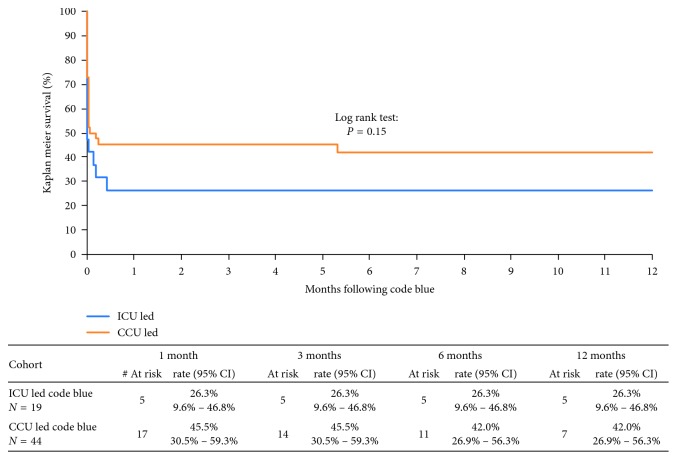
Survival rates for the ICU- vs. CCU-led study cohorts. CCU, Coronary Care Unit; ICU, Intensive Care Unit.

With respect to primary outcomes, a significantly greater proportion of patients survived the IHCA in the CCU-led cohort as compared to the ICU-led cohort (75% vs. 47%; *p*=0.03) (Table 1). In those individuals that survived the initial cardiac arrest, the hospital length of stay was longer at 32 days in the ICU-led group as compared to 9 days in the CCU-led group (Table 2). However, 1-year mortality was similar between both cohorts as shown in Figure 2. All 5 patients in the ICU-led cohort who survived their hospital stay demonstrated full neurological recovery. In contrast, 3 patients in the CCU-led cohort demonstrated neurological sequelae (2 delayed recoveries and 1 stroke) as shown in Table 2.

## 4. Discussion

As cardiovascular disease is a leading cause of morbidity and mortality worldwide, the management of individuals who sustain a cardiac arrest in the CCL remains a challenge. In the current study, we demonstrated that (1) the incidence of an IHCA within the CCL is low when acute coronary syndrome (ACS) was the primary indication for cardiac catheterization; (2) PEA was the most common rhythm for ACLS requiring chest compressions, with a mechanical device used in 25% of cases; (3) cardiogenic shock and arrhythmia were the most likely reasons for a code blue response to be initiated in the CCL; (4) neurological recovery among patients who survived an IHCA in the CCL is favourable; and (5) no significant difference was noted in the 1-year survival rate between the ICU-led and CCU-led cardiac arrest teams.

A review of the literature to date on PubMed/MEDLINE supports that the data on the prevalence of IHCAs in the CCL is limited. Webb et al. previously evaluated the prevalence of IHCAs in patients undergoing PCI at St. Paul's Hospital in Vancouver, BC, Canada, between 1996 and 1999 [8]. Of the total 4,363 patients (65 ± 13 years old, 61% males) who underwent PCI, only 1.3% suffered an IHCA in the CCL [8]. In addition, Sprung et al. conducted a retrospective analysis of all patients who suffered a cardiac arrest while undergoing coronary angiography or PCI at the Mayo Clinic between 1990 and 2000 [16]. Of the 51,985 coronary angiograms performed during this time frame, only 0.2% of patients suffered a cardiac arrest. Similarly, in our study, we evaluated the prevalence of IHCAs in the CCL from 2012 to 2016, during which time a total of 13,112 coronary angiograms were performed at our single tertiary care academic centre (∼2850 angiograms/year). Of this total cohort, only 0.5% suffered an arrest in the CCL necessitating ACLS. Despite the low prevalence of IHCAs in the CCL, patients who require an urgent coronary angiogram for ACS, cardiogenic shock, and/or OHCA may be at an increased risk of developing hemodynamic collapse necessitating ACLS during the invasive procedure [8, 16].

The initial electrical rhythm detected during a cardiac arrest in the CCL is predictive of survival. Previous studies have demonstrated that patients who suffer an IHCA with a shockable rhythm (VF and/or VT) have 3-4-fold improved survival rate, as compared to those patients presenting with a nonshockable PEA rhythm [17–21]. Our study confirmed PEA as the most common rhythm detected prior to the initiation of chest compressions in the CCL. This is likely a consequence of the success in achieving return of spontaneous circulation with the early recognition and defibrillation of ventricular arrhythmias in the CCL, during which time a code blue response was not necessarily activated.

In our study, a mechanical compression device was used in 25% of our patient population. The CCL is an ideal location for the use of a LUCAS device as trained staff can initiate the device early in ACLS, allowing the interventionalists to continue the cardiac catheterization procedure. Although previous multicentre studies have demonstrated that mechanical compression devices do not portend a mortality difference compared to conventional CPR, these studies only included individuals who suffered an OHCA [22–25]. As per the European Resuscitation Guidelines, early transition to the use of a mechanical chest compression device is strongly recommended in the CCL due to the difficulty of delivering manual chest compressions on the angiography table [20, 26–28].

An IHCA in the CCL is related to the severity and urgency of the patient's underlying ACS and in rare cases due to complications related to the PCI procedure itself. Webb et al. previously demonstrated that emergent coronary catheterization procedures constituted 2/3 of all cardiac arrests [8]. In their study, patients presenting with cardiogenic shock or with a STEMI/NSTEMI accounted for 72% and 60% of all cardiac arrests, respectively [8]. Only 29% of IHCAs that occurred in the CCL involved patients with stable or unstable angina [8]. Similarly, in our study, the majority (62%) of all cardiac arrests involved patients who presented with ACS or cardiogenic shock. This subset of individuals in cardiogenic shock requiring emergent angioplasty represents an unstable population at a high risk of cardiac arrest. On the contrary, a cardiac arrest secondary to a serious complication due to the cardiac catheterization procedure is rare. In our cohort, only 0.07% of patients undergoing a coronary angiogram suffered from an iatrogenic coronary artery dissection that resulted in a life-threatening event. This is consistent with the existing literature which reports an incidence of less than 0.1% [29].

There is a paucity of data addressing the neurological and long-term outcomes of individuals suffering a cardiac arrest in the CCL. In the Mayo Clinic study by Sprung et al., out of 51,985 coronary angiograms performed during 1990–2000, a total of 114 patients required CPR [16]. The investigators obtained a long-term follow-up for 64 patients who survived to hospital discharge, 58 were discharged home and 6 were admitted to personal care homes [16]. Full neurological recovery was noted in all patients at discharge. Our study corroborates the favourable neurological outcomes in >90% of patients surviving IHCA in the CCL. With regards to the 1-year survival rate, Sprung et al. reported a favourable rate of 80% in patients discharged from the hospital following IHCA in the CCL [16]. Wagner et al. noted a similar 1-year survival rate of 87% for discharged patients requiring mechanical chest compressions in the CCL between 2009 and 2013 [21]. Our current study noted a 100% 1-year survival rate for patients in both the ICU-led and CCU-led cohorts who survived to hospital discharge. Overall, 1-year survival rate from an IHCA in the CCL is high with favourable neurological outcomes.

Cardiologists are rarely involved in IHCA code blue teams, with ICU physicians primarily serving as team leaders [12, 13, 30]. A large observational study conducted in public, nonpsychiatric Danish hospitals evaluated all IHCAs between December 2012 and April 2013 [12]. This study observed that cardiologists are involved in only 27% of codes, with cardiology subspecialty trainees present at 5% of IHCAs. A study evaluating cardiac arrests at 32 hospitals in Rome, Italy, between 2000 and 2001 revealed an even lower participation of cardiologists in code blue teams at less than 20% [13]. Siebig et al. distributed a questionnaire to hospitals in Germany, Switzerland, and Austria to obtain data on the composition of resuscitation teams [30]. In their study, although the leader of the IHCA teams was identified as a noninternist/nonanesthesiologist in 4% of cases, they did not specify the proportion made up by cardiologists.

Given the underlying cardiac pathology of patients in the CCL, involvement of a cardiologist in the code blue team may offer a unique skillset, which includes cardiac rhythm analysis, bedside echocardiography, pericardiocentesis, and/or temporary pacing. In addition, continuous physiological monitoring in the CCL with ECG monitoring, pulse oximetry, invasive hemodynamics, immediate access to a mechanical compression device, and simultaneous PCI provides the unique opportunity to improve cardiac resuscitative efforts [9–11]. In the present study, a transitional change at our local tertiary institution placing the cardiologist as the leader of the code blue team in the CCL was instituted in 2014. As the 1-year survival rate was similar between the ICU-led and CCU-led groups, implementation of this role change in the evolving field of Critical Care Cardiology may be safe and effective.

There are several limitations associated with the current study. First, as a retrospective observational study, only documented IHCAs could be included in the analysis of outcomes. The disparity between the number of IHCAs occurring in the CCL between 2012–2014 (ICU-led cohort) and 2015–2016 (CCU-led cohort) raises suspicion that not all cardiac arrest events were documented. In addition, the two study cohorts were collected from different time points, before and after the implementation of an institutional change where the cardiologist was placed as the code blue team leader in the CCL. As such, the two patient populations were not identical, as reflected by the differences in smoking exposure, as well as the trend towards a greater proportion of NSTEMI or unstable angina cases in the ICU-led cohort. This has the potential to confound the survival impact observed from the change in the code blue team structure. Finally, the rarity of cardiac arrests in the CCL and subsequent small cohort size may have prevented the detection of differences in the overall survival rate of both cohorts.

## 5. Conclusion

Although the incidence of an IHCA in the CCL is low, individuals requiring emergent angioplasty for ACS or cardiogenic shock are at greater risk. In the evolving field of Critical Care Cardiology, the transition from an ICU-led to CCU-led code blue response team in the CCL setting may lead to similar short-term and long-term outcomes. Future clinical studies involving a larger patient population are required to confirm these findings.

## Figures and Tables

**Table 1:** A comparison of baseline characteristics in patients who had a cardiac arrest in the CCL led by the ICU versus the CCU teams, respectively. Data shown as percentage (absolute number) for all categorical variables. Coronary artery bypass grafting (CABG); mechanical circulatory support (MCS); peripheral vascular disease (PVD); pulseless electrical activity (PEA); ST elevation myocardial infarction (STEMI).

	Intensive Care Unit lead (2012–2014; *n* = 19)	Coronary Care Unit lead (2015-2016; *n* = 44)	*p* value
*Demographics*			
Age (years)	67 ± 10	70 ± 11	0.38
Sex (%, male)	73.7% (14)	54.6% (24)	0.15
Hypertension	57.9% (11)	61.4% (27)	0.80
Type 2 diabetes mellitus	26.3% (5)	27.3% (12)	0.94
Smoking history	52.6% (10)	18.2% (8)	<0.01
Hypercholesterolemia	42.1% (8)	34.1% (15)	0.54
PVD	26.3% (5)	13.6% (6)	0.28

*Etiology of code and duration of CPR*			
PEA arrest	68.4% (13)	36.4% (16)	0.02
Ventricular arrhythmia	15.8% (3)	27.3% (12)	0.52
Hypotension	15.8% (3)	36.4% (16)	0.10
Total CPR duration (min)	26.2 ± 8.2	28.5 ± 8.7	0.54
Standard CPR (min)	9.1 ± 2.3	5.6 ± 1.8	<0.01
Prolonged CPR (min)	31.2 ± 4.8	32.6 ± 4.1	0.64
Manual CPR (min)	21.8 ± 3.7	17.1 ± 2.8	0.25
Mechanical CPR (min)	29.8 ± 5.1	30.2 ± 4.7	0.70

*After code diagnosis*			
Coronary dissection	10.5% (2)	15.9% (7)	0.71
Cardiogenic shock	84.2% (16)	22.7% (10)	<0.01
Arrhythmia	5.3% (1)	38.6% (17)	<0.01

*Procedure post code*			
Intubation	68.4% (13)	58.1% (25)	0.44
Emergent pericardiocentesis	11.1% (2)	11.4% (5)	1.00
Angioplasty	61.1% (11)	52.3% (23)	0.53
CABG	22.2% (4)	6.8% (3)	0.18
MCS	38.9% (7)	20.5% (9)	0.13
Mechanical compression	26.3% (5)	25.0% (11)	<0.05
Device (LUCAS)	31.6% (6)	26.8% (11)	0.70

*Outcomes*			
Survived catheterization lab	47.4% (9)	75.0% (33)	0.03
Kaplan–Meier 1-year survival	26.3%	42.0%	0.15

**Table 2:** Comparison of secondary outcomes for patients surviving cardiac arrest in the ICU-led and CCU-led cohorts.

	Intensive Care Unit lead (Jan 2012–Dec 2014) (*n* = 19)	Coronary Care Unit lead (Jan 2015–Mar 2016) (*n* = 44)	*p* value
Acute kidney injury (survived cath lab)	14.3% (1)	21.1% (4)	1.00
Neurological Recovery			
Good	100% (19)	91.0% (40)	0.31
Delayed	0% (0)	4.5% (2)	1.00
Poor/stroke	0% (0)	4.5% (2)	1.00
Hospital length of stay in days (survived ICU stay)	32 (8–42)	9 (5–14)	0.32

## Data Availability

The data used to support the findings of this study are available from the corresponding author upon request.
